# Individualized hybrid electroconvulsive therapy (i-HECT) shows rapid anti-depressant effect in young patients with depression

**DOI:** 10.1186/s12888-026-08515-x

**Published:** 2026-08-11

**Authors:** Lun Zeng, Jing-ya Zhang, Jia Li, Mian-mian Chen, Shu-xian Xu, Chen-long Gu, Bai-jian Tan, Xin-hui Xie

**Affiliations:** 1 Department of Clinical Psychology, Second People’s Hospital of Huizhou, Huizhou, Guangdong China; 2 Electroconvulsive Therapy Room, Second People’s Hospital of Huizhou, Huizhou, Guangdong China; 3 Department of Psychiatry, Renmin Hospital of Wuhan University, Wuhan, Hubei China; 4 Department of Radiology, Washington University School of Medicine, St. Louis, MO USA; 5 Department of Psychiatric Rehabilitation, Second People’s Hospital of Huizhou, Huizhou, Guangdong China; 6 Department of Psychiatry, School of Clinical Medicine, University of Cambridge, Cambridge, CB2 0SZ UK

**Keywords:** Electroconvulsive therapy, i-HECT, Rapid antidepressant effect, Improved cognitive functions, Safety

## Abstract

**Background:**

For young patients with depression, electroconvulsive therapy (ECT) is highly effective but causes acute cognitive side effects. We designed a new individualized hybrid electroconvulsive therapy (i-HECT) combines ECT with low-charge electrotherapy (LCE) and individual symptom monitoring to reduce cognitive impairments.

**Methods:**

i-HECT comprised two treatments: ECT and LCE. ECT utilized an energy set of 1.5 times the seizure threshold (ST), while LCE was set at 0.5 ST. The initial session employed standard bilateral ECT. Subsequent sessions involved ECT or LCE, depending on whether meeting the ECT-LCE transition criteria (Montgomery-Åsberg Depression Rating Scale (MADRS) total score < 22 or ≥ 50% reduction), assessed after each session. This trial employed a Simon’s optimal two-stage design with a 3-month follow-up period in 46 young patients (age 16–25) with depression (MADRS ≥ 22).

**Results:**

The intention-to-treat analysis revealed an 80.4% response rate and a 58.7% remission rate, The reduction in MADRS score was 19.1 *±* 7.0, with Hedges’ *g* = 3.29 (95% CI = 2.66 to 3.91). The orientation recovery tests (ORTs) took 22.7 *±* 11.3 min after LCEs and 26.0 *±* 10.3 min after ECTs. Improvements in cognitive functions were observed after i-HECT treatments and during the 3-month follow-up periods.

**Conclusion:**

In conclusion, our i-HECT protocol shows promise as a simple and rapid treatment option for young patients with depression, with observed improvements in cognitive function.

**Trial registration:**

This trial was registered in the Chinese Clinical Trial Registry (http://www.chictr.org.cn), registration number: ChiCTR2100045682. Date of registration on April 23, 2021.

**Supplementary Information:**

The online version contains supplementary material available at https://doi.org/10.1186/s12888-026-08515-x.

## Introduction

Depression is one of the most common mental illnesses worldwide and a leading cause of disability [1, 2]. Epidemiological studies have shown that the lifetime prevalence of depression ranges from 8% to 12% [3, 4]. Among youth aged 14 to 25, suicide is the fourth leading cause of death in this age group, with depression being a major risk factor for suicide. Severe depressive symptoms in young patients can result in significant consequences, underscoring the critical need for prompt and efficacious antidepressant interventions. Electroconvulsive therapy (ECT), as a rapid treatment for depression, has demonstrated effectiveness in major depressive disorder (MDD), with a response rate of up to 75% within the first two weeks [5], while in psychotic depression the effectiveness reaches 90% [6]. Although ECT exhibits rapid antidepressant efficacy [7], it is still not widely accepted by young patients because of side effects, such as acute significant memory and cognitive impairments [8].

During ECT treatment, symptom reduction is rapid initially, and side effects increase over time. Many young patients, who are often in the academic phase of their lives, tend to choose some other physical treatment options, such as repetitive transcranial magnetic stimulation (rTMS), to minimize cognitive side effects and alleviate depressive symptoms. Although rTMS has a lower risk of severe cognitive side effects compared to ECT, its response rate (approximately 20–30%) is relatively lower and may require a longer treatment period [9–11]. Therefore, we need to develop an approach that can rapidly achieve antidepressant effects while minimizing side effects to maximize clinical benefits for young patients with depression. After conducting a literature review, reanalyzing existing clinical data, we found that the standard ECT treatment exhibits a “fast-then-slow” pattern in alleviating depressive symptoms; however, the cognitive-related side effects typically emerge after 3–5 treatments, displaying a “mild-then-severe” pattern [12–14]. In contrast, low-charge electrotherapy (LCE) which follows the same procedure as ECT but reduces the stimulus intensity to half the seizure threshold (ST), demonstrated a more linear symptom reduction rate, slower than the standard ECT in the initial treatments. However, LCE is associated with significantly fewer side effects [15, 16]. To balance efficacy and side effects, we proposed a new ECT strategy termed Hybrid-ECT (HECT) [17, 18]. In HECT, the first three sessions use standard bilateral ECT with energy set at 1.5 times the ST. Thereafter, it transitions to LCE at 0.5 ST. Our previous randomized controlled trial (RCT) demonstrated that HECT maintained similar antidepressant effects to ECT but with significantly fewer side effects [17].

Furthermore, after reviewing data from both HECTs and ECTs, we noted individual variations in the initial response to ECTs. For instance, while some patients experienced a 50% decline in depression scale scores after a single ECT session, others may require more than nine sessions to achieve similar results. The variability suggested the potential for personalized transition conditions from ECT to LCE within HECT, aiming to optimize therapeutic benefits. Therefore, we devised a new but simple protocol called individualized Hybrid-ECT (i-HECT). Under the i-HECT protocol, we assessed patients’ symptoms after each ECT/LCE session. If the predetermined ECT-LCE transition criteria were met, we implemented the switch. To evaluate the antidepressant efficacy and side effects (particularly cognitive side effects) of i-HECT in young patients with depression, we conducted this Simon’s optimal two-stage design trial.

## Methods

This single-arm trial employed a Simon’s optimal two-stage design [19] with a 3-month follow-up period, conducted at the Second People’s Hospital of Huizhou (April 2021–November 2023) in accordance with the Declaration of Helsinki [20]. Both patients and raters remained blinded to the ECT-LCE transition. Patients were not informed of the transition point at any stage of the trial, and staff maintained this blindness even if patients asked directly. Blinding was also maintained by physical and operational separation of the staff. Raters assessed participants in the ward, not in the ECT room. A research nurse recorded the orientation recovery time in a separate recovery room, and she remained unaware of the equipment setup in the ECT room. The Human Ethics Committee of the Second People’s Hospital of Huizhou approved the study protocol (approval number: 2021-010). Patients or their legal guardians provided informed consents and all participants or their legal guardians could withdraw from the trial at any time for any reason. Trial Registration: this trial was registered in the Chinese Clinical Trial Registry (http://www.chictr.org.cn), registration number: ChiCTR2100045682. Date of registration on April 23, 2021.

### Participants

Forty-six MDD/bipolar depression (BPD) patients were recruited based on the Inclusion criteria: (1) Age 16–25 years old; (2) Inpatients diagnosed with unipolar or bipolar depression according to ICD-10, with or without psychotic symptoms; (3) Baseline Montgomery-Åsberg Depression Rating Scale (MADRS) [21] score ≥ 22; (4) Treatment-resistant depression (TRD) or need for a rapid treatment response.

Exclusion criteria: (1) Pregnancy; (2) Participation in other studies within 30 days; (3) Undergoing of ECT or rTMS treatment within the past 6 months; (4) Previous poor response to ECT; (5) History of substance abuse disorder; (6) Presence of contraindications to ECT or anesthesia risks; (7) History of epilepsy.

### i-HECT procedures (see Fig. 1)

#### Basic settings

The i-HECT protocol comprised two treatments: ECT (1.5 ST) and LCE (0.5 ST), consistent with our previous report [17]. The dose titration procedure was performed to determine the ST during the first session. ECT/LCE sessions (three times per week) were performed using a spECTRUM 5000Q ECT instrument (MECTA Corporation, OR, USA), with pulse width of 1 ms and a fixed current of 800 mA. Anesthesia procedures included administration of etomidate (0.2–0.4 mg/kg), muscle relaxation with succinylcholine (1–2 mg/kg) and atropine (0–1 mg) depending on heart rate, and ventilation with 100% oxygen throughout the entire ECT/LCE procedures. Subsequent treatments were either ECT or LCE based on whether the patient met the criteria for ECT-LCE transition.

#### Criteria for ECT-LCE transition

The initial session was ECT, symptom assessments (MADRS) and brief cognitive evaluations (digital span and STROOP test) were conducted after each ECT/LCE session. When either of the following conditions was met, the next treatment switched to LCE: (a) MADRS total score < 22; (b) MADRS total score reduction ≥ 50%. Here, we adopted a MADRS score of 22 based on Leucht et al., who established a correspondence between a CGI-S score of “moderately ill” (score 4) and a MADRS range of 22–27 [22]. This score reflects a level of depression severity where transitioning to LCE may be appropriate while continuing to monitor progress.

#### Criteria for terminating i-HECT treatment

After meeting any of the following criteria, i-HECT treatment was terminated and transitioned to the follow-up period: (a) Completion of 12 ECT/LCE sessions; (b) MADRS total score *≤* 12; (c) A consecutive decrease of < 2 points over three MADRS assessments; (d) Other reasons, such as voluntary withdrawal or other medical considerations.

### Visit schedule

The five visits were set at (1) baseline (t0); (2) post-ECT-LCE-transition (t1); (3) post-treatment (within 24–48 h after the last ECT/LCE session) (t2); (4) end of the 1-month follow-up period (t3); and (5) end of the 3-month follow-up period (t4). Detailed visit schedule is shown in Table **S1**.

### Pharmacotherapy

Patients maintained their antidepressants and antipsychotics during the trial. Anticonvulsants, mood stabilizers, or lithium were discontinued during the i-HECT treatment. Single dose of alprazolam or oxazepam were prescribed as necessary when patients became agitated or felt anxious, but were prohibited 24-hour before ECT/LCE sessions. When patients suffered from insomnia, zopiclone, eszopiclone, or zolpidem were temporarily prescribed.

### Outcomes and statistical analysis

The primary outcome was the response rate analyzed in the intent-to-treat sample. Response was defined as a reduction of ≥ 50% in MADRS total score relative to baseline at the post-treatment visit. Additionally, achieving a MADRS score ≤ 12 was considered remission.

The trial utilizes an optimal Simon’s two-stage design [19] to assess the i-HECT response rate. This design strategy was chosen to maximize both ethical and resource efficiency by using an early stopping rule for futility to minimize the expected sample size, all while strictly adhering to the specified power (1 - β = 0.95) and type I error (α = 0.05). The core rationale for this design rests on two clinically relevant efficacy benchmarks: (1) Stage I: Null Threshold (*p*_*0*_ = 18.1%): This value does not define absolute ineffectiveness; rather, it establishes the lowest response rate considered insufficient to justify the next phase of research based on a risk-benefit assessment. We set *p*_*0*_ = 18.1% response rate of a widely used less-invasive brain stimulation alternative, TMS (O’Reardon et al.‘s RCT [10]). Since i-HECT involves higher invasiveness, anesthesia risk and more costs, it must demonstrate an efficacy superior to TMS to validate its clinical use. (2) Stage II: Alternative Threshold (*p*_*a*_ = 43%): This threshold represents the minimum clinical success required to proceed to a definitive, large-scale RCT. We set *p*_*a*_ = 43% based on the established response rate derived from a recently large-scale RCT for standard ECT (Kellner et al. [12]), which serves as the current clinical gold standard for refractory depression. Achieving this target confirms that i-HECT has sufficient promise to be tested against the current clinical gold standard. In stage I, nineteen patients will be recruited. If four or fewer responders are observed, the trial will be halted prematurely. Otherwise, an additional 27 patients will be recruited in stage II. If thirteen or more responses among these total 46 patients, the null hypothesis will be rejected, indicating promise for the i-HECT treatment.

The secondary outcomes including MADRS’s total scores and its subscales, 9-item Patient Health Questionnaire (PHQ-9) [23], Generalized Anxiety Disorder 7 (GAD-7) [24], Columbia–Suicide Severity Rating Scale (C-SSRS) [25], and Positive and Negative Syndrome Scale (PANSS) [26]. The subjective cognitive functions were evaluated using the Subjective Cognitive Decline Questionnaire (SCD-Q) [27]. The objective cognitive functions were measured using the Repeatable Battery for the Assessment of Neuropsychological Status (RBANS) [28], and Stroop Color and Word Test [29]. The orientation recovery tests (ORTs) were used to assess recovery time after each ECT/LCE session. Any adverse event (AE) or drop-out for any reason was recorded to analyze the safety. The total number of AEs per session was analyzed using a Poisson generalized linear mixed-effects model, then Individual adverse events were analyzed using binomial generalized linear mixed-effects models with a logit link as a sensitive analysis, ORT was analyzed using a linear mixed-effects model. The linear mixed model and the least square mean (LSM) and its 95% confidence intervals (CIs) were used to estimate the effects of outcomes at each visit using the *LmerTest* package [30] in R (version 4.2.0). The model included the visit, treated as a categorical variable, as a fixed effect and the subject as a random intercept.

Seizure-like electroencephalogram (EEG) duration was assessed using the following EEG-based criteria: 1. Electrographic Seizure: (a) Epileptiform discharges with an average frequency > 2.5 Hz persisting for > 10 s (> 25 discharges within 10 s); (b) Any EEG pattern demonstrating definite evolution, as defined above, and lasting > 10 s. 2. Electroclinical Seizure: Any abnormal EEG pattern, irrespective of frequency, that is accompanied by a clearly time-locked clinical correlate (e.g., subtle facial or limb twitching), without restriction on duration. 3. Sporadic Epileptiform Discharges: Occasional, transient epileptiform discharges, typically manifesting as spikes, spike-and-slow waves, or polyspike-and-slow waves, with a duration of < 10 s.

An exploratory subgroup analysis was conducted within the LCE phase to investigate the influence of seizure-like EEG duration on safety outcomes. LCE sessions were dichotomized into two groups based on the clinical standard for seizure adequacy: adequate seizure duration > 25 s) and non-adequate seizure duration (*≤* 25 s). We compared the incidence of AEs and ORT between these two subgroups.

## Results

### Participants

**Fig. 1:**
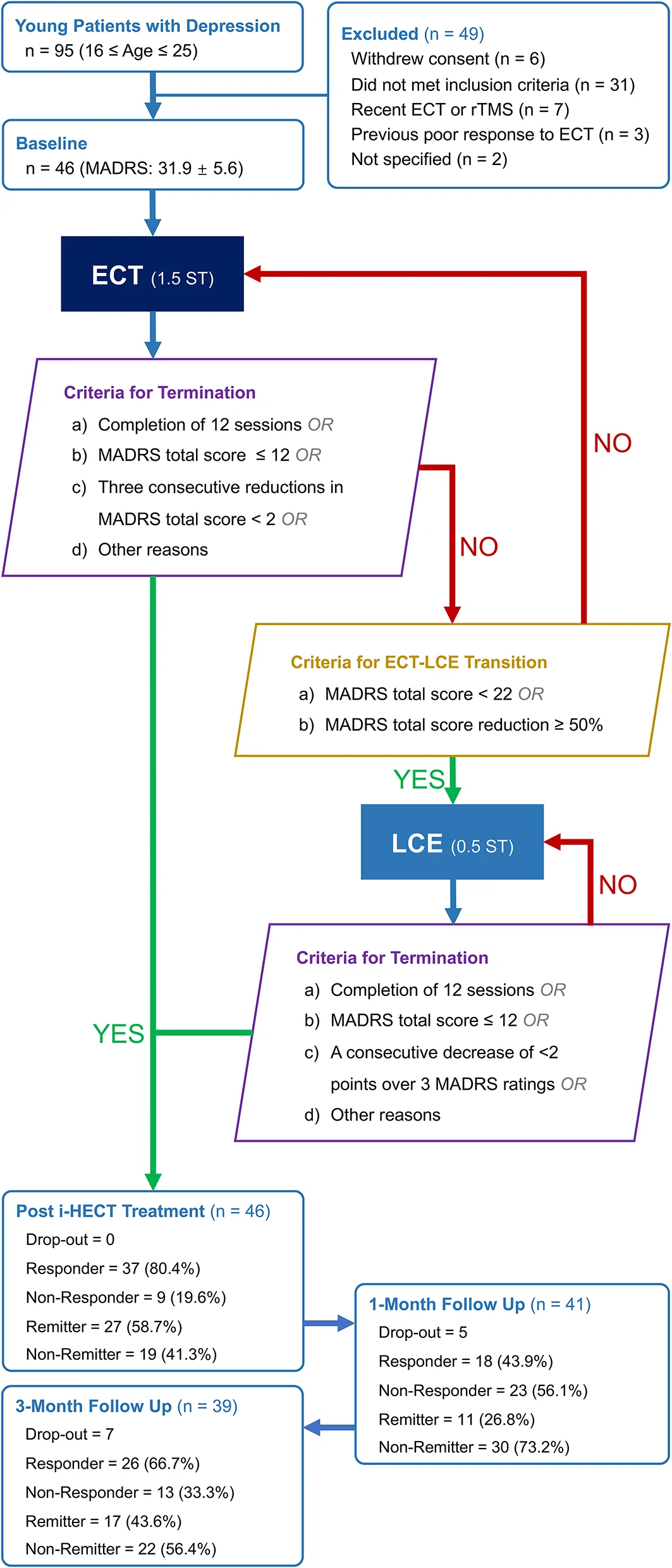
Flow chart. Abbreviations: ECT: electroconvulsive therapy; LCE: low-charge electrotherapy; ST: seizure threshold; rTMS: repetitive transcranial magnetic stimulation; MADRS: Montgomery-Åsberg Depression Rating Scale; i-HECT: individualized Hybrid-ECT

A total of 95 participants were screened, of which 49 were excluded upon enrollment. The flowchart is shown in Fig. 1.

Finally, 46 participants (32 females, mean age 18.7 *±* 2.3 years) were included in this trial; 58.7% of the sample was MDD and 41.3% was BPD. The mean baseline MADRS score was 31.9 (SD = 5.6) and details of the baseline characteristics are summarized in Table 1.

**Table 1:** Baseline characteristics

Characteristics	Results
Sex, Female, n (%)	32 (69.6%)
Age, year, mean ± SD	18.7 ± 2.3
Education Years, mean ± SD	11.8 ± 1.9
Diagnosis, MDD/BPD, n (%)	27 (58.7%)/19 (41.3%)
With Psychotic Feature, n (%)	19 (41.3%)
Onset Age, year, mean ± SD	15.8 ± 2.9
Disease Course, year, mean ± SD	2.7 ± 1.5
Current Episode, month, mean ± SD	3.8 ± 4.3
History of ECT, n (%)	5 (10.9%)
Equivalent Fluoxetine Dose, mg/day, mean ± SD	46.5 ± 16.2
Equivalent Olanzapine Dose, mg/day, mean ± SD	8.7 ± 3.8
Montgomery-Åsberg Depression Rating Scale (MADRS)	31.9 ± 5.6
Total Score, mean ± SD	
Cognitive Pessimism, mean ± SD	14.4 ± 2.3
Affective, mean ± SD	9.1 ± 2.3
Cognitive Anxiety, mean ± SD	5.1 ± 1.8
Vegetative, mean ± SD	6.2 ± 2.0
9-item Patient Health Questionnaire (PHQ-9), mean ± SD	19.3 ± 5.4
Generalized Anxiety Disorder 7 (GAD-7), mean ± SD	12.1 ± 5.6
Columbia–Suicide Severity Rating Scale (C-SSRS)	
Suicidal Ideation and Attempts, mean ± SD	3.5 ± 1.1
Intensity of Ideation, mean ± SD	16.3 ± 4.2
Stopped Suicide Attempt, mean ± SD	3.0 ± 1.6
Positive and Negative Syndrome Scale (PANSS)	
Positive Scale, mean ± SD	13.0 ± 2.3
Negative Scale, mean ± SD	17.9 ± 5.0
General Psychopathology Scale, mean ± SD	37.1 ± 5.4
Subjective Cognitive Decline Questionnaire, mean ± SD	14.4 ± 4.3
The Repeatable Battery for the Assessment of Neuropsychological Status (RBANS)	
Total Score, mean ± SD	396.1 ± 55.6
Immediate Memory, mean ± SD	70.8 ± 14.1
Visuospatial/Constructional, mean ± SD	83.4 ± 13.4
Language, mean ± SD	69.0 ± 18.5
Attention, mean ± SD	97.0 ± 14.4
Delayed Memory, mean ± SD	76.0 ± 19.6
Stroop Color and Word Test	
Error Interference Score, mean ± SD	2.0 ± 1.9
Time Interference Score, second, mean ± SD	39.1 ± 11.0

### Efficacy outcomes

**Fig. 2:**
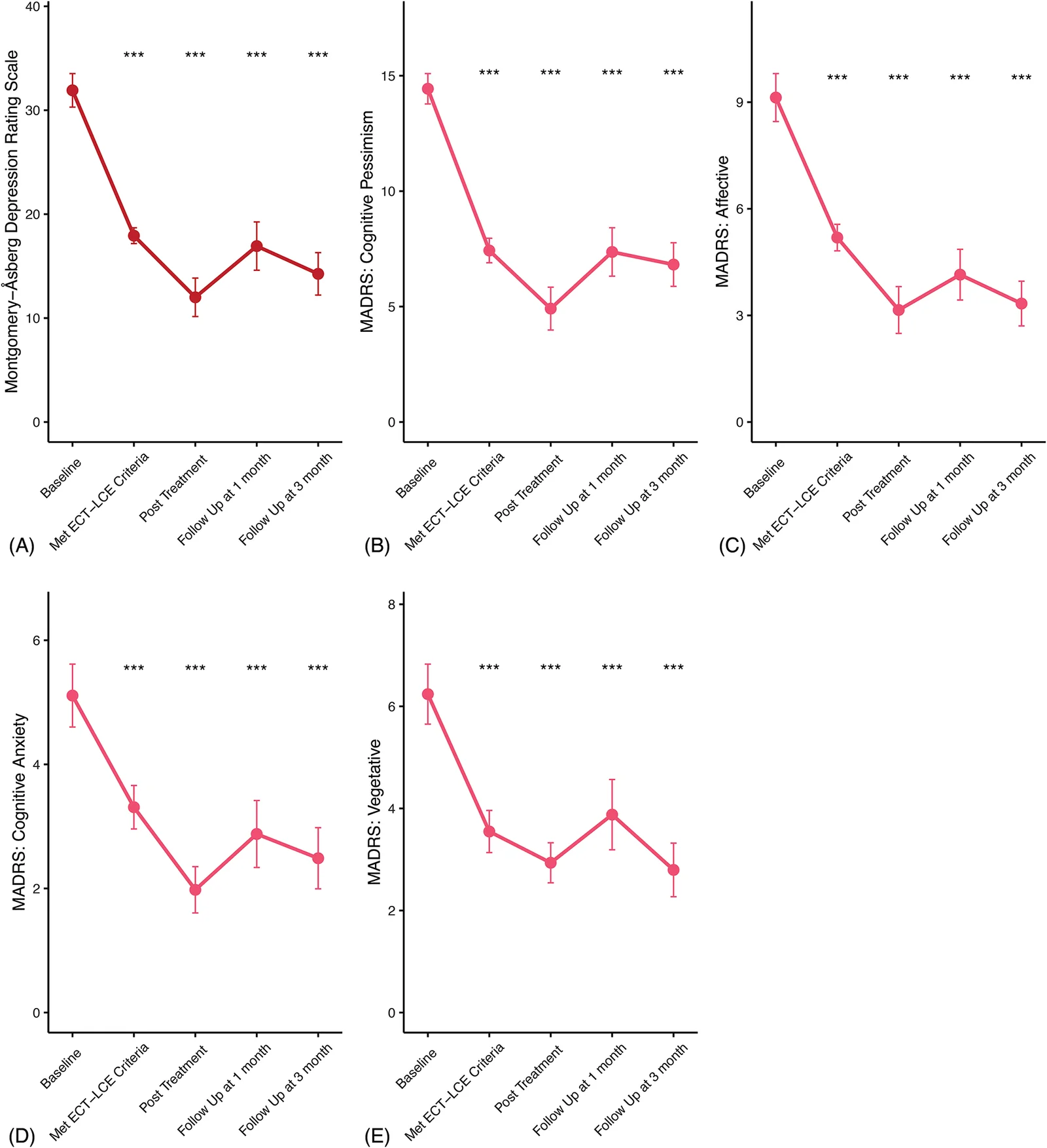
Changes in Montgomery-åsberg Depression Rating Scale (MADRS). Note: Error bars represent 95% confidence intervals (CIs). *p < 0.05, **p < 0.01, ***p < 0.001. Abbreviations: ECT: electroconvulsive therapy; LCE: low-charge electrotherapy

All 46 patients completed the i-HECT treatments. Five patients did not complete the 1-month follow-up for unspecified reasons, and seven patients did not complete the 3-month follow-up (Fig. 1). At the post-treatment visit, 37 patients (80.4%) achieved a response, with 27 (58.7%) achieving remission criteria. The mean number of treatment sessions was 6.0 *±* 2.1, with 3.0 *±* 1.8 sessions of ECT and 3.0 *±* 1.8 sessions of LCE. The stimulus doses for ECT and LCE were 80.0 *±* 29.1 mC and 30.7 *±* 11.2 mC, respectively (Wilcoxon rank sum test, *p* < 0.001). Seizures were also observed in a proportion of patients during LCE. Specifically, 42 patients exhibited one or more seizure-like EEG events during the LCE phase. The EEG-recorded seizure durations were 85.2 *±* 45.6 s during ECT and 75.7 *±* 66.5 s during LCE, respectively (Wilcoxon rank sum test, *p* = 0.046). Forty-three patients transitioned from ECT to LCE due to meeting the ECT-LCE transition criteria. Three patients did not meet the transition criteria and remained on ECTs throughout the trial, all the 3 patients were non-responders. The reduction in MADRS score was 19.1 *±* 7.0, with Hedges’ *g* = 3.29 (95% CI = 2.66 to 3.91). For details, please see Table 2  and Fig. 2. At the 3-month follow-up, i-HECT achieved response and remission rates of 66.7% and 43.6%, respectively.

**Fig. 3:**
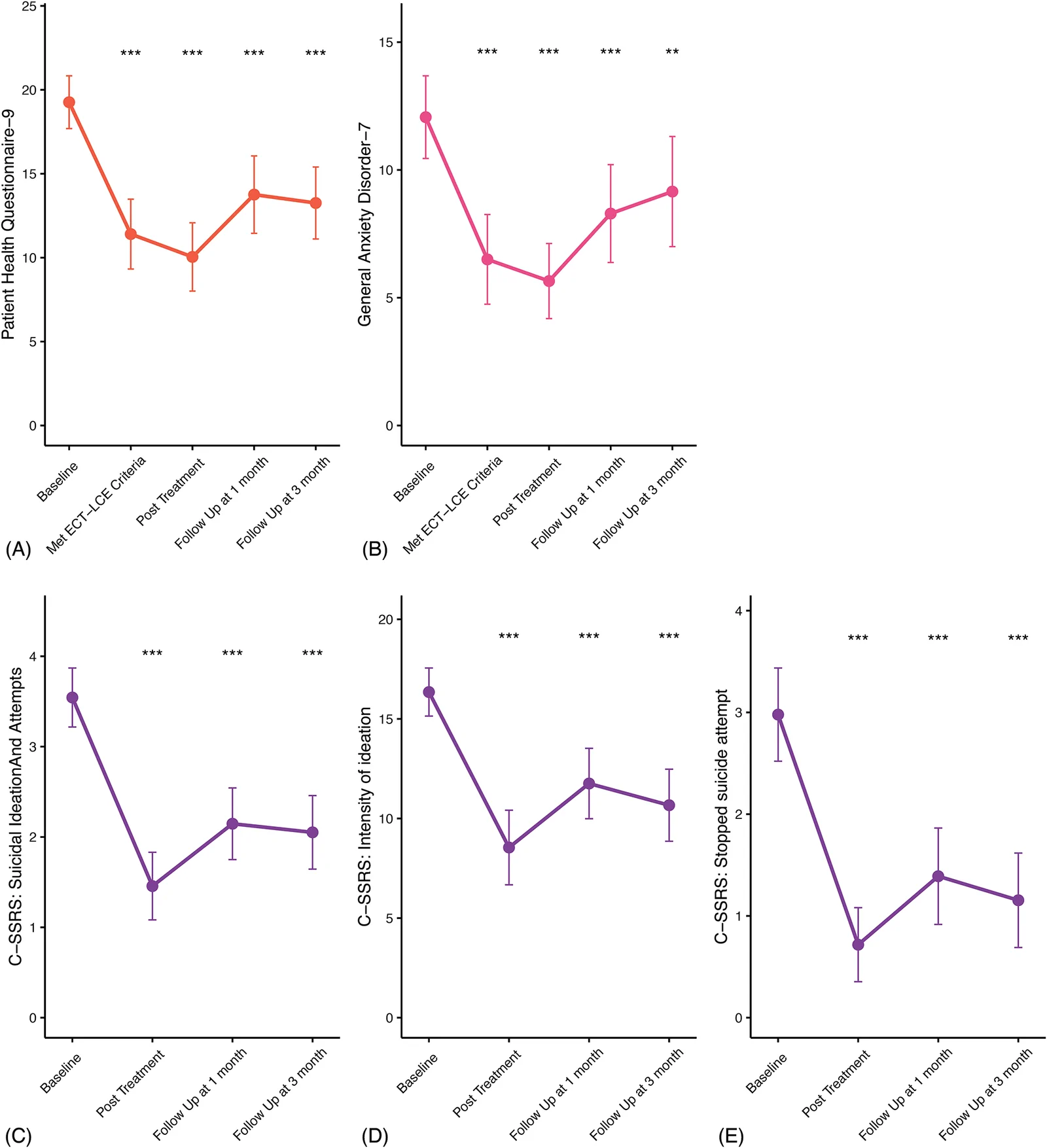
Changes in 9-item Patient Health Questionnaire (PHQ-9), Generalized Anxiety Disorder 7 (GAD-7), and Columbia–Suicide Severity Rating Scale (C-SSRS). Note: Error bars represent 95% confidence intervals (CIs). *p < 0.05, **p < 0.01, ***p < 0.001. Abbreviations: ECT: electroconvulsive therapy; LCE: low-charge electrotherapy

**Fig. 4:**
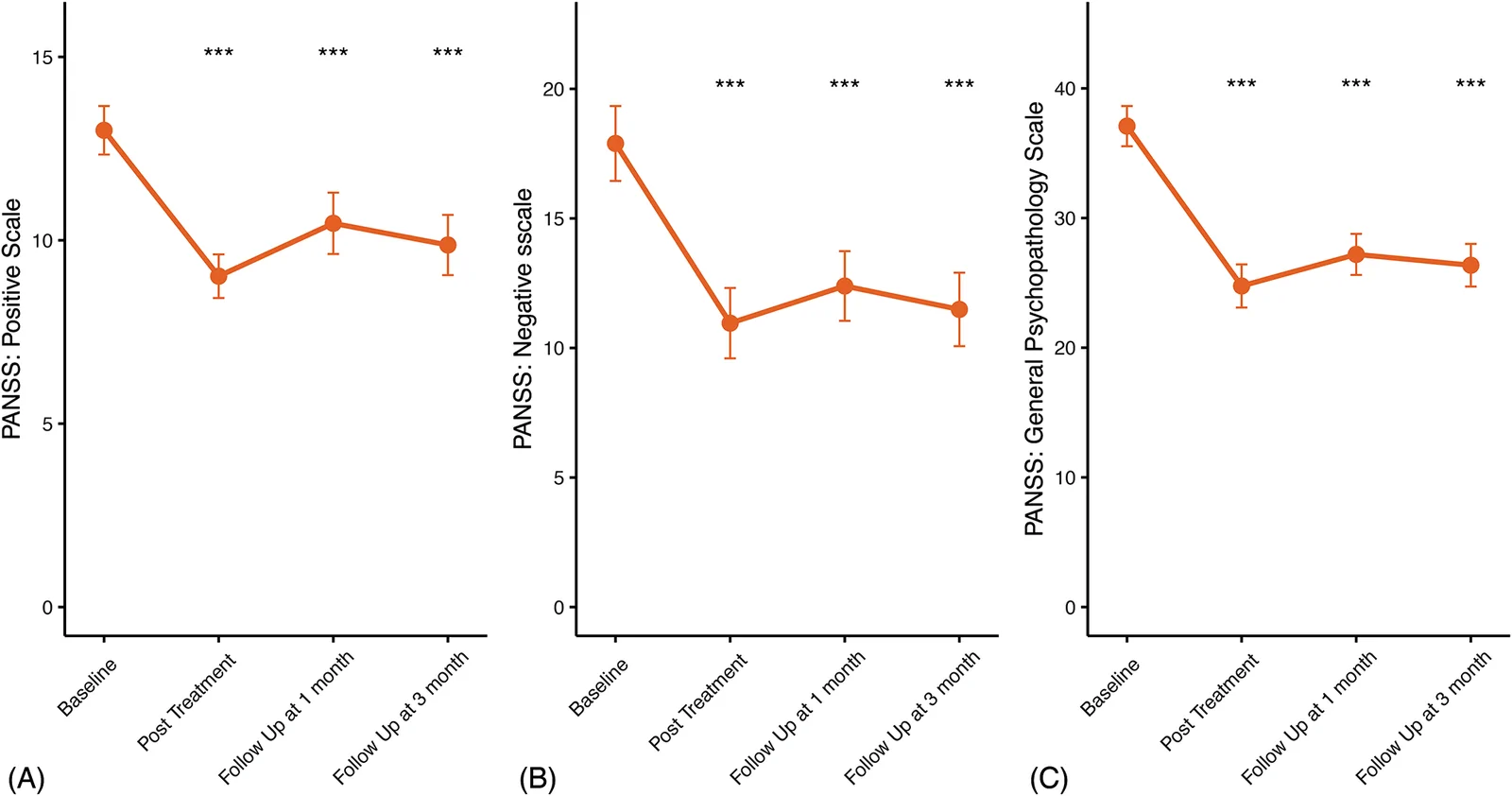
Changes in Positive and Negative Syndrome Scale (PANSS) Note: Error bars represent 95% confidence intervals (CIs). *p < 0.05, **p < 0.01, ***p < 0.001. Abbreviations: ECT: electroconvulsive therapy; LCE: low-charge electrotherapy

Following i-HECT treatments, significant improvements were observed across various measures including MADRS, PHQ-9, GAD-7, C-SSRS, and PANSS (Table 2; Figs. 3 and 4). Throughout the follow-up period, clinical management primarily consisted of maintenance pharmacotherapy. Notably, no participants received additional sessions of either standard ECT or LCE during this phase.

**Table 2:** Mixed-model repeated measures analysis of clinical and cognitive outcomes

Outcomes	Visit	Change From Baseline (95% CI)	*p*
**Montgomery-Åsberg Depression Rating Scale (MADRS)**			
Total Score	Met ECT-LCE Criteria (*n* = 42)	-13.4 (-15.4 to -11.4)	< 0.001
	Post Treatment (*n* = 46)	-19.9 (-21.8 to -18)	< 0.001
	One Month Follow Up (*n* = 41)	-14.6 (-16.6 to -12.6)	< 0.001
	Three Month Follow Up (*n* = 39)	-17.4 (-19.4 to -15.4)	< 0.001
Cognitive Pessimism	Met ECT-LCE Criteria	-6.8 (-7.8 to -5.8)	< 0.001
	Post Treatment	-9.5 (-10.5 to -8.5)	< 0.001
	One Month Follow Up	-6.9 (-7.9 to -5.9)	< 0.001
	Three Month Follow Up	-7.5 (-8.5 to -6.5)	< 0.001
Affective	Met ECT-LCE Criteria	-3.7 (-4.5 to -3)	< 0.001
	Post Treatment	-6 (-6.7 to -5.3)	< 0.001
	One Month Follow Up	-4.9 (-5.6 to -4.2)	< 0.001
	Three Month Follow Up	-5.7 (-6.5 to -5)	< 0.001
Cognitive Anxiety	Met ECT-LCE Criteria	-1.7 (-2.2 to -1.1)	< 0.001
	Post Treatment	-3.1 (-3.7 to -2.6)	< 0.001
	One Month Follow Up	-2.2 (-2.7 to -1.6)	< 0.001
	Three Month Follow Up	-2.6 (-3.2 to -2.1)	< 0.001
Vegetative	Met ECT-LCE Criteria	-2.6 (-3.3 to -2)	< 0.001
	Post Treatment	-3.3 (-3.9 to -2.7)	< 0.001
	One Month Follow Up	-2.3 (-3 to -1.7)	< 0.001
	Three Month Follow Up	-3.4 (-4.1 to -2.7)	< 0.001
**9-item Patient Health Questionnaire (PHQ-9)**			
	Met ECT-LCE Criteria (*n* = 42)	-7.4 (-9.3 to -5.5)	< 0.001
	Post Treatment (*n* = 46)	-9.2 (-11.1 to -7.3)	< 0.001
	One Month Follow Up (*n* = 41)	-5 (-7 to -3.1)	< 0.001
	Three Month Follow Up (*n* = 39)	-5.9 (-7.8 to -3.9)	< 0.001
**Generalized Anxiety Disorder 7 (GAD-7)**			
	Met ECT-LCE Criteria (*n* = 42)	-5.3 (-7 to -3.5)	< 0.001
	Post Treatment (*n* = 46)	-6.4 (-8.1 to -4.7)	< 0.001
	One Month Follow Up (*n* = 41)	-3.5 (-5.3 to -1.8)	< 0.001
	Three Month Follow Up (*n* = 39)	-3 (-4.8 to -1.2)	0.002
**Columbia–Suicide Severity Rating Scale (C-SSRS)**			
Suicidal Ideation and Attempts	Post Treatment (*n* = 46)	-2.1 (-2.5 to -1.7)	< 0.001
	One Month Follow Up (*n* = 41)	-1.3 (-1.8 to -0.9)	< 0.001
	Three Month Follow Up (*n* = 39)	-1.5 (-1.9 to -1)	< 0.001
Intensity of Ideation	Post Treatment	-7.8 (-9.6 to -6)	< 0.001
	One Month Follow Up	-4.3 (-6.2 to -2.4)	< 0.001
	Three Month Follow Up	-5.6 (-7.6 to -3.7)	< 0.001
Stopped Suicide Attempt	Post Treatment	-2.3 (-2.8 to -1.8)	< 0.001
	One Month Follow Up	-1.5 (-2 to -1)	< 0.001
	Three Month Follow Up	-1.8 (-2.3 to -1.3)	< 0.001
**Positive and Negative Syndrome Scale (PANSS)**			
Positive Scale	Post Treatment (*n* = 46)	-4 (-4.8 to -3.2)	< 0.001
	One Month Follow Up (*n* = 41)	-2.5 (-3.3 to -1.7)	< 0.001
	Three Month Follow Up (*n* = 39)	-3.2 (-4 to -2.4)	< 0.001
Negative Scale	Post Treatment	-6.9 (-8.2 to -5.7)	< 0.001
	One Month Follow Up	-5.3 (-6.6 to -4)	< 0.001
	Three Month Follow Up	-6.2 (-7.6 to -4.9)	< 0.001
General Psychopathology Scale	Post Treatment	-12.3 (-14 to -10.7)	< 0.001
	One Month Follow Up	-9.5 (-11.2 to -7.8)	< 0.001
	Three Month Follow Up	-10.6 (-12.3 to -8.9)	< 0.001
**Subjective Cognitive Decline Questionnaire**			
	Met ECT-LCE Criteria (*n* = 42)	-1.9 (-3.3 to -0.5)	0.007
	Post Treatment (*n* = 46)	-3.3 (-4.7 to -1.9)	< 0.001
	One Month Follow Up (*n* = 41)	-2.7 (-4.1 to -1.2)	< 0.001
	Three Month Follow Up (*n* = 39)	-3.5 (-4.9 to -2.1)	< 0.001
**The Repeatable Battery for the Assessment of Neuropsychological Status (RBANS)**			
Total Score	Met ECT-LCE Criteria (*n* = 37)	32.9 (18.9 to 47)	< 0.001
	Post Treatment (*n* = 46)	58.4 (45.3 to 71.4)	< 0.001
	One Month Follow Up (*n* = 41)	57.7 (44.1 to 71.3)	< 0.001
	Three Month Follow Up (*n* = 39)	70.5 (56.7 to 84.3)	< 0.001
Immediate Memory	Met ECT-LCE Criteria	13.7 (8.8 to 18.6)	< 0.001
	Post Treatment	23.5 (18.9 to 28)	< 0.001
	One Month Follow Up	25.5 (20.8 to 30.2)	< 0.001
	Three Month Follow Up	29 (24.2 to 33.8)	< 0.001
Visuospatial/Constructional	Met ECT-LCE Criteria	3.5 (-1.2 to 8.3)	0.145
	Post Treatment	6.3 (1.9 to 10.7)	0.006
	One Month Follow Up	2 (-2.6 to 6.6)	0.392
	Three Month Follow Up	5.2 (0.5 to 9.9)	0.03
Language	Met ECT-LCE Criteria	5.6 (1 to 10.2)	0.018
	Post Treatment	9 (4.7 to 13.3)	< 0.001
	One Month Follow Up	11.6 (7.2 to 16.1)	< 0.001
	Three Month Follow Up	11.8 (7.3 to 16.3)	< 0.001
Attention	Met ECT-LCE Criteria	3 (-1.5 to 7.5)	0.195
	Post Treatment	4.3 (0.1 to 8.5)	0.046
	One Month Follow Up	4.2 (-0.2 to 8.6)	0.061
	Three Month Follow Up	8.3 (3.8 to 12.8)	< 0.001
Delayed Memory	Met ECT-LCE Criteria	9 (4.5 to 13.5)	< 0.001
	Post Treatment	15.3 (11.1 to 19.5)	< 0.001
	One Month Follow Up	14.7 (10.3 to 19)	< 0.001
	Three Month Follow Up	16.4 (12 to 20.8)	< 0.001
**Stroop Test**			
Error Interference Score	Met ECT-LCE Criteria (*n* = 42)	-1.1 (-1.5 to -0.6)	< 0.001
	Post Treatment (*n* = 46)	-1.2 (-1.7 to -0.8)	< 0.001
	One Month Follow Up (*n* = 41)	-1 (-1.4 to -0.5)	< 0.001
	Three Month Follow Up (*n* = 39)	-1.4 (-1.8 to -0.9)	< 0.001
Time Interference Score	Met ECT-LCE Criteria	-4.3 (-6.9 to -1.8)	0.001
	Post Treatment	-7.3 (-9.8 to -4.8)	< 0.001
	One Month Follow Up	-2.4 (-5 to 0.2)	0.073
	Three Month Follow Up	-6.6 (-9.2 to -4)	< 0.001

### Safety

No serious adverse events were reported during the study. In the total sample, the most commonly reported AEs were memory complaints, headaches, and fatigue. The Poisson generalized linear mixed-effects model showed that, after adjustment for treatment visit and within-participant repeated measurements, the total number of adverse events per session was significantly lower during LCE than during ECT (*p* = 0.010), corresponding to an approximately 34% reduction. Among the individual AEs, the adjust odds ratios (ORs) for most events were lower during LCE than during ECT; however, statistically significant reductions were observed only for headache (*p* < 0.001) and fatigue (*p* = 0.003). ORT was significantly shorter during LCE than during ECT, with an adjusted mean difference of -3.84 min (*p* = 0.033) (Table 3).

**Table 3:** Safety results

	ECT (*N* = 137)	LCE (*N* = 137)	Effect measure, estimate (95% CI)^a^	*p*
**Adverse event (AE)**				
Total number of AEs per session, Mean *±* SD	2.3 *±* 1.6	1.3 *±* 1.5	IRR: 0.66 (0.48 to 0.91)	0.010
Headache, n (%)	61 (44.5%)	20 (14.6%)	OR: 0.16 (0.06 to 0.46)	< 0.001
Fatigue, n (%)	55 (40.1%)	24 (17.5%)	OR: 0.20 (0.07 to 0.58)	0.003
Nausea, n (%)	12 (8.8%)	3 (2.2%)	OR: 0.94 (0.10 to 8.63)	0.957
Euphoria, n (%)	8 (5.8%)	7 (5.1%)	OR: 2.36 (0.39 to 14.4)	0.354
Confusion, n (%)	5 (3.6%)	2 (1.5%)	OR: 0.49 (0.01 to 48.3)	0.762
Memory Complaint, n (%)	86 (62.8%)	65 (47.4%)	OR: 0.80 (0.27 to 2.36)	0.681
Dry Mouth, n (%)	23 (16.8%)	19 (13.9%)	OR: 0.70 (0.15 to 3.25)	0.645
Muscle Pain, n (%)	17 (12.5%)	8 (5.8%)	OR: 0.69 (0.15 to 3.25)	0.638
Dizziness, n (%)	42 (30.7%)	20 (14.6%)	OR: 0.38 (0.12 to 1.14)	0.085
Irritability, n (%)	9 (6.6%)	13 (9.5%)	OR: 1.08 (0.19 to 6.26)	0.927
**Orientation Recovery Time (ORT)**				
Minute, Mean *±* SD	26.0 *±* 10.3	22.7 *±* 11.3	AMD: -3.8 (-7.4 to -0.3)	0.033

Note: a: The analysis was conducted among 42 participants who received both ECT and LCE; data from four participants who received ECT only were excluded from the analysis. Abbreviations: AE, adverse event; SD, standard deviation; ECT, electroconvulsive therapy; LCE, low-charge electrotherapy; IRR, incidence rate ratio; OR, odds ratio; AMD, adjusted mean difference; CI, confidence interval

The exploratory analysis of the LCE phase (summarized in Table**S2**) revealed that the occurrence of an adequate seizure was not significantly associated with the incidence of common AEs. However, LCE sessions without adequate seizure durations were associated with a significantly shorter ORT compared to those with adequate seizures (*p* = 0.007).

### Cognitive function

**Fig. 5:**
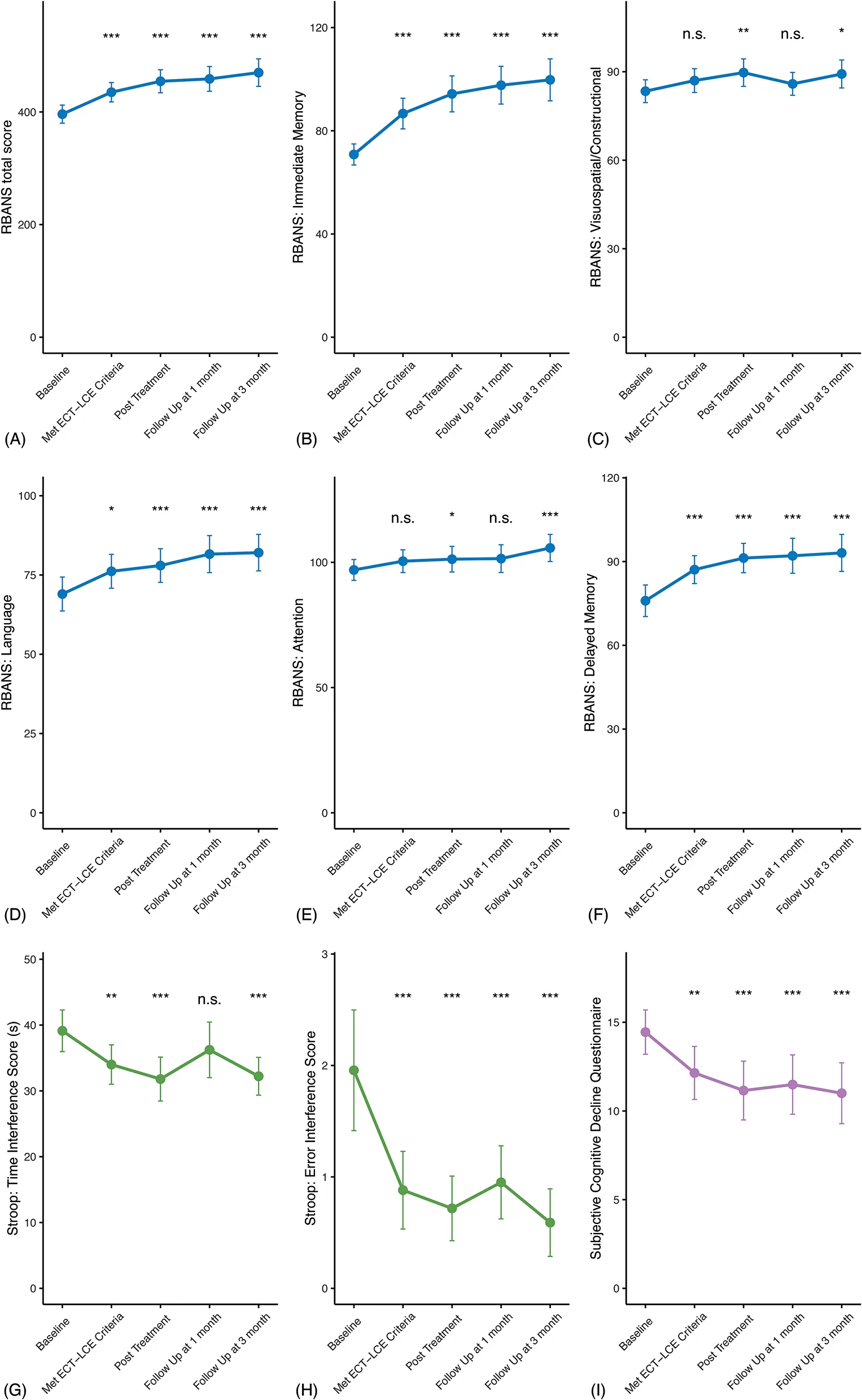
Changes in Objective (RBANS and Stroop tests) and Subjective Cognitive (SCD-Q) Functions Note: Error bars represent 95% confidence intervals (CIs). *p < 0.05, **p < 0.01, ***p < 0.001. Abbreviations: RBANS: Repeatable Battery for the Assessment of Neuropsychological Status. SCD-Q: Subjective Cognitive Decline Questionnaire; ECT: electroconvulsive therapy; LCE: low-charge electrotherapy

Notably, cognitive function performance, a key focus of this trial, showed significant improvements in both subjective and objective assessments. As shown in Table 2; Fig. 5, the subjective cognitive function impairments were measured by SCD-Q, the score at the post-treatment was significantly lower than baseline and continued to decrease during the 3-month follow-up (*p* < 0.001).

The total scores of the RBANS and the subscale (including Immediate Memory, Visuospatial/Constructional, Language, Attention and Delayed Memory) scores were significantly higher at the end of the i-HECT treatment compared to baseline, and these improvements were maintained during the 3-month follow-up period.

Regarding executive function, both reaction times and the number of response errors on the Stroop test were significantly lower than baseline levels.

### Data accessibility

All individual participant data (IPD) are presented in Table S3.

## Discussion

In brief, this trial successfully met its primary endpoint. The observed response rate (80%) not only rejected the pre-specified null hypothesis of uninterestingness (*p*_*0*_ = 18.1%) but also exceeded the promising threshold (*p*_*a*_ = 43%) derived from standard ECT data. This positive finding indicates that i-HECT is a promising intervention and provides a strong rationale for a future, large-scale, head-to-head RCT to formally compare its efficacy and safety against standard ECT. Moreover, the most promising outcome was the significant improvement observed in both subjective and objective cognitive functions post-treatment, with this trend continuing during the 3-month follow-up period.

We also observed improvements in both subjective and objective cognitive functions post-treatment, with this trend continuing during the 3-month follow-up period following the i-HECT protocol. However, this finding must be interpreted with caution. As has been well-established, post-treatment cognitive functioning in depressed patients is strongly associated with the level of symptom improvement [31, 32]. We cannot conclude that this cognitive improvement is a direct effect of i-HECT, nor that i-HECT is superior to standard ECT in this regard. Although a previous RCT [17] from our group found that a slightly different protocol (HECT, which, in contrast to the i-HECT in this study, involved a fixed switch to LCE after 3 ECT sessions) was associated with better cognitive outcomes than standard ECT, the current single-arm study lacks a comparator group, and we cannot disentangle the effects of treatment from those of symptom remission. Therefore, the observed cognitive improvements in this study should be viewed as preliminary. They demonstrate the potential of this protocol and provide important preliminary data for our planned, future three-arm RCT, which will be designed to formally compare the cognitive effects of i-HECT, HECT, and standard ECT.

Young patients with depression are typically in early stages of their education or career, making prompt intervention imperative. While ECT is a most evidence-supported rapid antidepressant treatment, concerns about cognitive side effects have reduced its use in this population of young patients with depression. However, our i-HECT protocol may provide a new and simple option for addressing these concerns in these young individuals.

The most crucial metrics for evaluating a therapeutic intervention are its response and remission rates. However, these indicators largely depend on the clinical characteristics of patients, such as age, treatment resistance, and psychotic symptoms, etc [33]. To appropriately assess the potential of i-HECT, our results must be compared with those of ECT studies conducted in similar populations (i.e., young patients or adolescents). A study on young people with treatment-resistant depression reported a response rate of 85.4% and a remission rate of 29.3% [14]. Another study on adolescents with severe mood disorders found a response rate (CGI *≤* 2) of 52.8% after ECT [34]. A study involving young adults and adolescents with depression showed that the response rate and remission rate of young adults were 86.5% and 45.6% respectively, while those of adolescents were 65% and 28.1% respectively [35]. The participants in this study were relatively young (average age 18.7 *±* 2.3 years), and 19 individuals (41.3%) with psychotic depression, which are strong predictors of good ECT outcomes [33]. Against this backdrop, the post-treatment response rate of 80% and remission rate of 60% achieved by i-HECT in this study are comparable to those of similar studies mentioned above. Additionally, given that i-HECT is an adaptation of HECT, our previous RCT reported response and remission rates for HECT at 73.3% and 26.7%, respectively. Furthermore, i-HECT maintained a response rate of 66.7% and a remission rate of 43.6% at the 3-month follow-up. Our finding on relapse is similar to previous research, which reported that approximately half of all patients successfully treated with ECT relapse within one year, with most relapses occurring within the initial six months, even when receiving maintenance pharmacotherapy [36]. Our result generally aligns with this established context. Nevertheless, we look forward to larger studies providing a more accurate estimation of the true relapse rate. Overall, these findings suggest that i-HECT holds promise for young patients, especially those with psychotic features, and justifying the need for further RCTs.

A potential theoretical concern regarding our protocol is that the fixed transition threshold (MADRS < 22) might lead to a switch from standard ECT to LCE after only a marginal improvement. For instance, a patient with a baseline score of 23 could theoretically transition following a mere 1-point reduction. However, the mean baseline MADRS score in our cohort was 31.9 *±* 5.6, suggesting that for the majority of patients, the transition required a substantial clinical change. To further address this concern, we reviewed our individual participant data (IPD). No patients in this cohort had a baseline score of exactly 22, and only four patients (8.7%) started with a score of 24. A detailed review of these four cases confirmed that their transitions were clinically meaningful: two patients improved by more than 6 points after the first session, while the other two required multiple ECT sessions to achieve a significant score reduction (reaching scores of 15 and 17, respectively) before meeting the switch criteria. These practical trajectories suggest that the 22-point threshold is a reliable indicator of clinical improvement in this population. Nevertheless, we acknowledge that this criterion could be further refined in future large-scale studies to ensure the optimal timing for the ECT-to-LCE transition.

A particularly noteworthy finding is that i-HECT demonstrated robust outcomes across various cognitive assessments, especially in short-term cognitive performance, contrasting with the common perception of standard ECT. Patients undergoing standard ECT frequently reported side effects such as cognitive impairments, headaches, and disorientation [37]. These cognitive deficits were typically restricted to the first three days post-treatment and tended to resolve within two weeks [38]. In contrast, our trial findings highlight the following: First, regarding AEs, patients receiving i-HECT primarily reported headaches and fatigue, but these AEs mainly occurred during the ECT treatment phase. Second, in terms of cognitive performance, both subjective cognitive reports and objective cognitive assessments showed that most cognitive functions recovered rapidly during the treatment process and were maintained at the 3-month follow-up. Our trial also observed shorter ORT during the LCE phase, which may be due to the lower charge used. Similarly, other research suggests that ORT indirectly predicts past personal memory loss [39]. This finding may also relate to the cognitive performance in our study. However, because this was a single-arm trial, we cannot draw conclusions about the causal relationship among these three factors. We look forward to testing this possibility in RCTs. Moreover, i-HECT does not require modifications to ECT devices; significant benefits can be achieved through simple adjustments to the ECT dosage strategy. This characteristic facilitates the broader application of i-HECT therapy.

Another critical consideration is that the efficacy and cognitive side effects of ECT are influenced by parameters such as electrode placement, pulse width, and electrical dosage [12, 40, 41]. While right unilateral (RUL) placement is generally associated with fewer cognitive side effects compared to bitemporal (BT) or bifrontal (BF) placements [42–44], it is often accompanied by a bit slower onset of antidepressant effects [12]. In our trial, achieving a rapid antidepressant response early in treatment is essential. This urgency is particularly evident when combined with LCE, which seeks to provide swift symptom relief while prioritizing cognitive preservation. In this trial, patients who received i-HECT showed marked early antidepressant improvements and notable improvements in cognitive measures. Future research will focus on exploring the potential efficacy of alternative electrode placements during the initial phase of i-HECT.

Surprisingly, even with the much lower stimulus charge used during LCE, many patients still developed EEG-recorded seizures. These seizures were shorter and more variable than those seen in standard ECT. The necessity of seizures in ECT has been debated in recent years. Since 1938, seizures have traditionally been viewed as essential for therapeutic effects. Some studies report a positive link between seizure duration and antidepressant response [45]. For instance, Cronholm and Ottosson found that patients with shorter seizures required more treatments and had poorer outcomes than controls [46]. While we cannot determine the true relationship between seizures and efficacy now, we cannot rule out that the seizures during LCE sessions played an anti-depressive role. Our LCE subgroup analysis further clarifies the multifaceted dynamics of acute cognitive recovery in the i-HECT protocol. The observation that AE incidence was independent of seizure adequacy suggests that the reduced electrical charge may be the primary driver in minimizing overall physical discomfort and cognitive burden. Meanwhile, the significantly shorter ORT observed in LCE sessions without adequate seizures indicates that seizure duration specifically influences the rate of immediate postictal reorientation. These results highlight the necessity for future studies to more precisely calibrate the interplay between electrical dosage and seizure characteristics to further optimize the i-HECT paradigm.

On the other hand, some researchers propose that cognitive side effects of ECT may be related to the high electrical charge. In clinical practice, clinicians often use 1.5–2.5 times the seizure threshold to ensure a robust seizure. Peterchev and colleagues suggest that the large electrical dose may be a major cause of cognitive side effects, and they therefore explored reducing and individualizing current amplitude in ECT [47]. Lower and more individualized stimulation may generate seizures closer to the neural activation threshold and make the induced seizure more focal, which may reduce cognitive adverse effects [47, 48]. Magnetic seizure therapy (MST), which has gained increasing attention in recent years, also supports this idea. MST produces seizures but has fewer cognitive side effects than ECT. Because magnetic fields penetrate scalp and skull more easily, MST can target brain regions more precisely. MST-induced seizures start in superficial cortical areas, while ECT affects deeper structures such as the hippocampus, which may contribute to cognitive side effects [49, 50]. Studies comparing MST and ECT also show differences in EEG seizure expression: MST produces weaker theta, alpha, and beta activity during seizures, less post-ictal suppression, and a faster recovery profile [51, 52]. These findings suggest that better control of the electric field may reduce the intensity and spread of seizures while preserving clinical efficacy.

Taken together, these studies show that electrical stimulation and seizures are closely related and cannot be viewed in isolation. Sub-threshold stimulation without seizures tends to have limited antidepressant effects, while excessive charge used to induce strong seizures may produce significant side effects. Therefore, achieving the best balance, using an appropriate electrical dose to induce an appropriate seizure may be the key to maximizing treatment benefits while minimizing adverse effects for patients with depressive disorders. Furthermore, combining the observations from this trial with our previous RCT [17], it seems plausible that the roles of charge and seizure are not constant across different treatment stages. There may be an ECT-LCE trade-off point that helps balance rapid gains with the smallest possible side effects.

### Limitations

First, as this study was a single-arm trial without a control group, standard ECT group, or sham ECT group, while we observed outcomes such as response and remission rates, these effects cannot be solely attributed to i-HECT, as external factors, including the placebo effect, may have influenced the results. Second, although the trial showed promising clinical outcomes, especially in cognitive improvements, we cannot conclusively assert that i-HECT is significantly superior to standard ECT. These results are better considered as preliminary, signal-generating, and associative, rather than confirmatory. Further, we should also consider that the observed cognitive improvements could reflect a reduction in depressive symptoms, repeated testing effects, or general recovery over time. Because of experimental limitations, this trial cannot determine whether cognition improved due to i-HECT itself or these other factors. Nevertheless, the findings provide a strong foundation for conducting a more rigorous RCT and offer valuable insights to inform its design. Third, although the safety comparison between the ECT and LCE phases was based on within-participant repeated measurements and analyzed using mixed-effects models, the treatment phases were not randomized or counterbalanced. ECT was administered before LCE according to the i-HECT protocol. Therefore, the observed differences may have been influenced by treatment order, improvement in depressive symptoms, adaptation to repeated treatment and anaesthesia, or other time-varying factors. The lower AE burden and shorter ORT during LCE should therefore be interpreted as adjusted within-participant associations rather than definitive evidence of a causal safety advantage over standard ECT. These findings require confirmation in a randomized head-to-head trial. Fourth, the trial enrolled young patients, which may reduce potential heterogeneity due to age diversity but also limited the generalizability of the results to other age groups. Future research should include participants across a wider age range to ensure a more comprehensive understanding. Fifth, during the LCE phase, seizure-like EEG events of variable durations were observed, ranging from brief episodes (< 10 s) to prolonged events exceeding 25 s and, in some cases, over 100 s. The shorter events may reflect neuronal activation induced by electrical stimulation that was insufficient to elicit a fully developed (adaptive) seizure. However, due to the constraints of the study design, we are unable to determine the underlying mechanisms or the precise clinical significance of these seizure-like EEG events during the LCE phase. Also, seizure threshold was determined only before the first treatment session and was not reassessed during the treatment course, as repeated threshold titration has limited feasibility in routine clinical practice. Based on the available data, we are unable to determine why seizure-like EEG events were recorded during the later LCE phase. We have therefore reported these observations as they were recorded, without proposing a definitive explanation. Sixth, patients remained on antidepressants and antipsychotics, whereas lithium, mood stabilisers, and anticonvulsants were discontinued during i-HECT. This protocol complicated the assessment of both efficacy and cognitive changes. The withdrawal of certain medications alongside the continuation of others served as a potential source of confounding, making it difficult to isolate the precise therapeutic and adverse effects of i-HECT. As this study represents the first clinical trial of i-HECT, we have made the complete raw dataset publicly available to facilitate subsequent hypothesis-driven investigations and to provide a foundation for independent replication and further research.

## Conclusions

In conclusion, our i-HECT protocol shows promise as a simple and rapid treatment option for young patients with depression. The observed cognitive improvements suggest that this protocol warrants further evaluation in randomized controlled trials to determine its direct clinical efficacy.

## Supplementary Information

Below is the link to the electronic supplementary material.

Supplementary Material 1: Table S1. Visit Procedure.docx

Supplementary Material 2: Table S2. Differences_Between_Seizures_during_LCEs.xlsx

Supplementary Material 3: Table S3. Individual_Participant_Data.xlsx

## Data Availability

All individual participant data (IPD) are presented in Table 3 .

## Abbreviations

AE: Adverse event
BF: Bifrontal
BPD: Bipolar depression
BT: Bitemporal
CI: Confidence interval
C-SSRS: Columbia–Suicide Severity Rating Scale
EEG: Electroencephalogram
ECT: Electroconvulsive therapy
GAD-7: Generalized Anxiety Disorder 7
i-HECT: Individualized hybrid electroconvulsive therapy
IPD: Individual participant data
LCE: Low-charge electrotherapy
LSM: Least square mean
MADRS: Montgomery-Åsberg Depression Rating Scale
MDD: Major depressive disorder
PANSS: Positive and Negative Syndrome Scale
PHQ-9: 9-item Patient Health Questionnaire
RBANS: Repeatable Battery for the Assessment of Neuropsychological Status
rTMS: Repetitive transcranial magnetic stimulation
RCT: Randomized controlled trial ()
SCD-Q: Subjective Cognitive Decline Questionnaire
ST: Seizure threshold
