# Supplementary material for: Individualized hybrid electroconvulsive therapy (i-HECT) shows rapid anti-depressant effect in young patients with depression
Source: BMC Psychiatry. 2026 Aug 11;26:880. doi: 10.1186/s12888-026-08515-x (PMC13746425; doi:10.1186/s12888-026-08515-x)
Supplement: Supplementary file 1 [file 12888_2026_8515_MOESM1_ESM.docx]

sTable X. Visit schedule

|  | baseline | After each session | post-ECT-LCE-transition | post-treatment | 1-month follow-up | 3-month follow-up |
| --- | --- | --- | --- | --- | --- | --- |
| Visit | t0 |  | t1 | t2 | t3 | t4 |
| MINI | X |  |  |  |  |  |
| PHQ-9 | X |  | X | X | X | X |
| GAD-7 | X |  | X | X | X | X |
| PANSS | X |  | X | X | X | X |
| MADRS | X | X | X | X | X | X |
| Stroop test | X | X | X | X | X | X |
| Digital span | X | X | X | X | X | X |
| C-SSRS | X |  | X | X | X | X |
| SCD-Q | X |  | X | X | X | X |
| RBANS | X |  | X | X | X | X |
| AEs |  | X |  |  |  |  |
| ORT |  | X |  |  |  |  |

PHQ-9: 9-item Patient Health Questionnaire, *GAD-7*: Generalized Anxiety Disorder 7, *PANSS*: Positive and Negative Syndrome Scale, *MADRS*: Montgomery-Åsberg Depression Rating Scale, *C-SSRS*: Columbia–Suicide Severity Rating Scale, *SCD-Q*: Subjective Cognitive Decline Questionnaire, *RBANS*: Repeatable Battery for the Assessment of Neuropsychological Status, *AE*: adverse events, *ORT*: Orientation recovery test
